# Sanitary Condition of the Mouth and Teeth, with a Business Method of Obtaining the Same

**Published:** 1900-08

**Authors:** Levi C. Taylor

**Affiliations:** Hartford, Conn.


### SANITARY CONDITION OF THE MOUTH AND TEETH, WITH A BUSINESS METHOD OF OBTAINING THE SAME.[1]

BY DR. LEVI C. TAYLOR, HARTFORD, CONN.

The suggestions offered for your consideration this evening are based upon principles and facts with which you are all more or less familiar, but I desire to make their practical value more apparent.

Webster defines the term *dentist* as "One who cleans, extracts, repairs, or fills natural teeth and inserts artificial ones." The later editions conclude by pronouncing him a *dental surgeon.*

Harris's Dictionary, of 1867, under the head of Dental Surgeon, says, "He is one who devotes himself to the study and treatment of the diseases of the teeth and their connections."

The last definition suits our purpose, as it better describes the thought when we refer to the sanitary condition of the mouth.

That there is disease in a very large percentage of mouths at the present time it is not probable any will attempt to deny. Whether the cause is higher civilization, the use of soft foods, or is produced by a large combination of circumstances, we will not here attempt to discuss.

That it is the duty of us who assume the responsibility, to care for the mouths of our patients in the best manner, we all believe. It seems fitting, as honest and conscientious men, that we should

---

[1] Read before the Academy of Dental Science, May 2, 1900.

be well prepared, and up to the teachings of our best minds. We should seek to establish a scientific truth for ourselves.

Science Dr. Elliott would probably define as "systematized facts."

If we find one or more facts that can be demonstrated at all times, we have a right to speak of the discovery as scientific; and such I believe to be the case in the practically new method of dealing with our patients.

In the teachings of the late Dr. Riggs, thirty and even forty years since, he said that if we would clean teeth well enough and as often as circumstances required, there would be no decay. Dr. Riggs was a man of more professional than business ability; consequently, he almost failed to procure for his patients the valuable results his ability seemed to indicate.

Our Andrews, our Black, and our Americo-English Williams have repeatedly shown us on the screen many fine pictures of the microbes that exist in the mouths of the human family, proving beyond question that they are present in great abundance. As far as I am aware none have given a practical suggestion how to rid ourselves of the injury of such infections. While we are fully convinced that such myriads of germ life exist, it is very apparent they are fed and nourished to a more mature form by the numerous irritations that are allowed to remain on and around the teeth. The erosion then forms sufficient to give them a good foothold beneath the enamel and until the little tendrils are sent out penetrating the tubuli. The general health structure is broken down, and then we have what is called decay.

Treating decay has attracted our attention for many years, probably being, in most men's hands, more remunerative than the earlier prophylaxis, which should be considered for the good of our patients, and, when considered rightly, may be pronounced for our good. When our patients derive a benefit at our hands, they are usually willing to share that benefit with him who is the giver.

I wish here to comment on the teachings of our schools. The first year is devoted to anatomy, physiology, and chemistry, with such infirmary practice as the cleaning of teeth. I will venture to say that the students of many schools do not average to clean one set of teeth a week, and many not more than two or three during the term.

Right here allow me to ask the question, How much time is devoted to the proper teaching of the sanitary condition of the mouth? Ask yourselves this question, How much time was devoted by any member of the faculty or superintendent of the infirmary when you were a student? and you will be surprised at the inattention, aside from the occasional assignment of a patient, allowing you to practise as best you could.

Few of the schools seem to pay much, if any, attention to this beyond the first year. The second year the student has attained sufficient dignity to make amalgam fillings, and the third year he has reached the golden age. The fourth year he has obtained his degree, but is not supposed to consider such a humble occupation as the cleaning of teeth, except when called upon especially for such service, when the teeth are whipped over five or ten minutes with the engine, and the doctor passes on for another golden nugget. I once heard a D.D.S. remark to a student that "Any fool could clean a set of teeth." I very soon learned that he who made the remark had never learned the first rudiments of cleaning teeth with any thought for the sanitary effects. He simply rubbed off the faces that they might look a little better. Such cleaning is absolutely harmful, for it deceives both patient and dentist and leads them to think they have the teeth cleaned while all the harmful part is left lurking between the teeth and under the gums, where it is best suited to do its deadly work of destruction.

I believe in the modern dental engine, and consider it an absolute necessity for many parts of our work, but I have never seen a case bearing good results in the line of thorough cleaning of the mouth when it was used. It is simply a delusion, for it does the work open to the eye with such apparent satisfaction that we fail to go up under the gum with our orange-wood and pumice to complete the operation beyond criticism. Hand-cleaning has been proven to be the only thorough method in that line.

As regards the time necessary for cleaning a full set of teeth, thirty minutes is the minimum, when everything is favorable. As the complications increase, it will extend into hours of hard, laborious, nerve-exhausting work, for which the practitioner should be compensated at a higher fee per hour than for ordinary filling, as it is many times more valuable when considered from a pathological stand-point than any of the more common operations.

It seems desirable that our teachers of pathology should extend their time and instruction to the infirmary, and there demonstrate the results that may be obtained from modern prophylaxis.

Many of our practitioners get much information (such as it is) from the drummer who has learned for what purpose a particular instrument is designed, and he imparts such knowledge as best he can, that he may extend his sales. We are all more or less afflicted with the sample fiend, who has some wonderful germicide which he wishes us to try, guaranteeing that it will cure every known disease. He is not content with the declaration that the remedy will remove the tartar and green coating on the teeth, but he goes further, and says it will cure every known form of toothache.

How far the hypnotic suggestions of our sample men have aided in the efforts that are being put forth for more perfect sanitary results I am not able to judge. That there is such effort being made by many and in many different ways I do believe.

It is costing the profession two generations of persistent instruction to counteract the effects of such teaching as was demonstrated by the ancient practitioner, whose sole remedy for all pathological disturbances was the forceps.

Many people to-day are wearing artificial dentures, greatly to their discomfort, as the result of improper instruction given their ancestors, possibly two or three generations ago.

Here I wish to state that it is much more difficult to fully vindicate our prophylaxis theory when a partial plate is worn, as so few will keep the plates and teeth as clean as is desirable.

You will notice I have not referred to this matter under the head of Riggs's disease, or pyorrhœa. It is better to stimulate an interest in the prophylaxis theory; and as each of us becomes thoroughly enthused over the results of these cases which we are *able to make successful,* we will feel our way on into the advanced cases with a better understanding of how far we can attain success. Too many failures have been made already by attempting to care for cases in advance of our actual knowledge as a pathologist or manipulator.

Dr. D. D. Smith, of Philadelphia, has taken great interest in producing the best results for his patients. He has been experimenting for six years along the line of prophylaxis, with such success as to have convinced several of our Philadelphia friends, who have told me personally that what he claims is true, for they have demonstrated his theory for themselves.

Dr. Smith has written some excellent articles on the subject of prophylaxis, one of which was published in the INTERNATIONAL DENTAL JOURNAL and afterwards copied into the *Dental Digest.* Another is soon to appear in the *Dental Digest.* It is not my purpose to dwell on what is contained in that article, for you can have the pleasure of reading it yourselves. It was my privilege, a little more than a year ago, to stand beside the dental chair of Dr. Smith and to have him explain each one of many cases which have been under his care for several years. I noted the practical methods that were used to bring about most excellent results.

I was interested, yes, delighted, with what I saw and heard.[1]

For more than thirty years I have been a great admirer of fine dentistry, especially the general results that have been attained, but never have I seen anything like this.

Dr. Smith informed me that he had never been able to fully obtain such results until of late years, for he was never able to have the full control of the mouth, as, he said, patients would at various times allow little matters to defer their appointments indefinitely, until the benefits he so much desired were lost.

He conceived the idea of making a yearly contract with the patient for a certain sum of money to be paid annually whether present or not, the only stipulation being that the patients should present themselves regularly each month for him to polish and massage the teeth thoroughly, at his discretion, he guaranteeing immunity against decay,—the mouth being put in perfect condition at the outset,—working on the theory that when the tooth surface is well polished no decay will take place.

The massaging will so stimulate the teeth that it in part makes up for the lack of use which nature designs.

After seeing these things, and the results that followed, I became so elated that on my return I took two of the worst mouths I had in my practice and commenced seeing the patient each month, following along the line suggested by Dr. Smith, and the results are more gratifying than I had dared hope for.

One patient told me, the other day, that she had never been half the time before without having teeth filled, and, said she, "I

---

[1] One good Quaker lady leaned forward, as the doctor was showing her mouth, and told me, with the usual Quaker modesty, "It is the best thing I have found in dentistry."

do not dread to come to see you any more than I dread to go down-town shopping."

The other patient told me that she really enjoyed having her teeth massaged and polished.

I now have some fifteen patients on this basis, several of whom would be willing to pay double the fee charged rather than go back to former methods.

The gums become a beautiful, healthy pink, rigid and hard, and you can crowd up under them with the orange-wood and pumice almost harshly without discomfort to or resistance from the patient.

The wisdom of this practice becomes more apparent every day, and with such success attending it, is it not reasonable that one becomes enthusiastic?

